# Development of nasopharyngeal carcinoma target delineation: from two-dimensional radiotherapy to adaptive precision radiotherapy

**DOI:** 10.7150/jca.125480

**Published:** 2026-06-04

**Authors:** Xiaojing Yang, Lijun Liu, Huicai Zhang, Hanru Ren, Jie Fu

**Affiliations:** 1 Department of Radiation Oncology, Shanghai Sixth People's Hospital Affiliated to Shanghai Jiao Tong University School of Medicine, Shanghai, China; 2 Department of Orthopedics, Shanghai Pudong Hospital, Pudong Medical Center, Fudan University, Shanghai, China.; 3 Division of Urology, Department of Surgery, Brigham and Women's Hospital & Harvard Medical School, Boston, MA, USA.

**Keywords:** Nasopharyngeal carcinoma, Radiotherapy, Target delineation

## Abstract

Nasopharyngeal carcinoma (NPC) is mainly treated with radiotherapy. With the advancements of imaging and radiotherapy techniques, the delineation of NPC target areas has evolved from empirical field setting in the two-dimensional era to three-dimensional conformal radiotherapy (3D-CRT), intensity modulated radiotherapy (IMRT), and image-guided adaptive radiotherapy (ART), significantly improving treatment accuracy and safety. This review systematically explores the development process of target area delineation in NPC, focusing on the evolution and standardization of target areas in the nasopharynx and neck lymph nodes, and combines the role of multimodal imaging such as MRI and PET-CT in target area delineation. The article further examines the clinical response strategies and research trends of target area changes after induction chemotherapy (IC), and looks forward to the potential of ART and artificial intelligence in future target area delineation. The review aims to provide clinical practitioners with a scientific, rational, and personalized approach to target delineation, optimizing treatment plans and improving clinical outcomes.

## Introduction

Nasopharyngeal carcinoma (NPC) is an epithelial derived malignant tumor originating from the nasopharyngeal mucosa, exhibiting distinct epidemiological, pathological, and biological characteristics 1. Its incidence rate has obvious geographical distribution characteristics, which is relatively high in southern China (especially Guangdong and Guangxi), Southeast Asia, the Middle East and North Africa, and is closely related to EB virus infection, genetic factors and environmental exposure 2, 3.

Radiotherapy is the most important and effective curative treatment for NPC, especially for patients in stages I-III, offering favorable local control and long-term survival outcomes 4, 5. The success of modern radiotherapy depends not only on the optimization of technical equipment and treatment plans, but also on the accurate delineation of the target area. Reasonable target delineation can minimize normal tissue damage while ensuring tumor control, thereby reducing complications and improving patients' quality of life (QoL) 6.

With the continuous advancement of imaging technologies such as CT, MRI, and PET-CT, radiotherapy technology has also developed from two-dimensional radiotherapy (2D-RT) to three-dimensional conformal radiotherapy (3D-CRT), intensity modulated radiotherapy (IMRT), image-guided radiotherapy (IGRT), and adaptive radiotherapy (ART) 7-9. The target area delineation is gradually transitioning from "empiricism" to "evidence driven". Especially in recent years, with the widespread application of induction chemotherapy, tumor volume and morphology have undergone significant changes 10, 11, posing new challenges and adjustment strategies for target delineation.

This article intends to start with the target delineation during 2D-RT, summarize the technological evolution of NPC delineation, and focus on exploring the target delineation standards for nasopharyngeal and cervical lymphatic drainage areas in modern radiotherapy. Combined with multimodal image fusion technology, adjustment strategies after induction chemotherapy, and the forefront of future adaptive and intelligent radiotherapy, it provides reference for the formulation of clinical treatment plans and standardized target tumor delineation.

## Target delineation in the era of 2D-RT

In the era of 2D-RT, radiotherapy plans are mainly based on anteroposterior and lateral X-ray images, and the treatment field setting heavily relies on anatomical experience and traditional standards 12. The irradiation range of the primary lesion of NPC usually includes: upper boundary: the upper edge of the sella turcica or the base of the frontal sinus; lower bound: level of hyoid bone or cricoid cartilage; front boundary: posterior wall of nasal cavity; posterior boundary: the first cervical vertebra or slope 13. Cervical lymphatic irradiation usually uses contralateral or three fields, covering the I-V area, with an irradiation range from the skull base to the supraclavicular fossa 14, without clear imaging support.

NPC patients had relatively low local control and survival rates due to technological limitations in the early era of 2D-RT. A retrospective study in Hong Kong analyzed NPC patients (n = 2687) who received radiotherapy from 1996 to 2000. Among them, 90% of patients received 2D-RT. It was found that their 5-year overall survival (OS) rate was only 75%, and the local recurrence rate was as high as 15% or more, especially in T3-T4 stage patients 15. A study on the coverage of NPC target area by 2D-RT showed a 5-year local recurrence free rate (LRFS) of 66%. The gross tumor volume (GTV) was covered by 95% isodose lines in only 9% of patients 14. Another study from Hong Kong showed that under a 2D-RT regimen, the average maximum doses to the brainstem and spinal cord were 61.8 Gy and 56 Gy, respectively, both exceeding the conventional dose limits of 54 Gy for the brainstem and 45 Gy for the spinal cord 16. Over-irradiation of normal tissues may result in significant toxicity burden. These data provide a realistic foundation and comparative background for the subsequent application of 3D technology and IMRT.

Challenges in the 2D-RT era include blurred target boundaries, inaccurate tumor delineation leading to under- or overexposure, and significant radiation toxicity to normal tissues like the brainstem, cochlea, and spinal cord. Additionally, 2D-RT fails to account for tumor heterogeneity and individual anatomical variations.

## 3D-CRT and IMRT

The popularization of CT simulation positioning has led radiotherapy from empirical field setting to image-guided approach 17. Through 3D reconstruction, physicians can identify tumor boundaries and draw accurate GTV. This stage marks the introduction of the concepts of CTV (clinical target volume) and PTV (planning target volume), and the shift of radiotherapy dose from a single plane to volume control.

IMRT regulates the shape and intensity of the radiation beam through a multi-directional multi leaf collimator, achieving "dose carving" while balancing high-dose tumor coverage and organs at risk (OAR) 18. RTOG 0225 and 0615 studies constructed a standard target area stratification for NPC: GTVnx: visible portion of primary lesion image; GTVnd: lymph node metastasis with clear imaging; CTV1 (high-risk area): GTVnx extends 5-10 mm, covering the pharyngeal opening of the Eustachian tube, posterior nasal orifice, upper edge of the hard palate, and so on; CTV2 (medium low risk area): II-IV and other lymphatic drainage areas, with lower doses (54-60 Gy) 19, 20. This scheme has become a classic reference for IMRT target delineation.

IMRT greatly improves the treatment efficacy of NPC. A retrospective study of 847 NPC patients treated with IMRT radiotherapy showed a 5-year LRFS rate of 94.3% 21, far superior to historical 2D-RT data. Additionally, a multicenter study involving 22,302 NPC patients found a 2.68% incidence of radiation-induced cranial nerve paralysis and a median survival of 13.2 years, confirming IMRT's safety and effectiveness 22. Massachusetts General Hospital has proposed an individualized CTV delineation scheme for NPC, which involves reducing the target area, including only delineating the ipsilateral pharyngeal space, irregularly including the nasal/maxillary sinus or ethmoid sinus, not delineating the pharyngeal airway, and irregularly delineating the sphenoid sinus. The 5-year local control rate was 94%, and all four patients who experienced local recurrence were GTV intratumoral recurrence 23. Their results indicate that narrowing the target area can achieve good local control, minimal toxic reactions, and no field recurrence. State Key Laboratory of Oncology in Southern China conducted MRI monitoring of local progression patterns in 943 NPC patients, and developed a new personalized CTV plan based on the results: CTV1 is GTV external placement of 5-10mm, including the entire nasopharyngeal mucosa and pharyngeal fascia structure; CTV2 includes all bilateral high-risk areas (parapharyngeal space, posterior nasal cavity, pterygoid process, anterior vertebral muscles, clitoris, petrous apex, hiatus, base of sphenoid bone) 24. Sun Yat-sen University Cancer Center has refined the CTV delineation plan for early NPC T1 and T2 staging, reduced the range of CTV, decreased the radiation dose, and adopted an accelerated large segment radiotherapy plan (GTVp: 68Gy/30F). Follow up for 10 years showed that this plan achieved high local control rate and overall survival rate 25. For patients with unilateral NPC without contralateral posterior pharyngeal lymph node metastasis or EBV-DNA positivity, studies have shown that CTV range can be reduced (reducing the contralateral posterior upper wall, but including the contralateral pharyngeal recess) 26. Cancer Hospital of Fujian Medical University analyzed 414 cases of "small target area CTV" scheme, and found that the boundary distance of GTV-CTV2 in locally advanced (T3, T4) patients was smaller compared to the average level of all patients, but the local control rate of the tumor did not significantly decrease. A new CTV target area delineation approach was proposed: CTV = GTV + certain external edges + entire nasopharyngeal mucosa 27. The research results on the application of improved reduced IMRT in NPC showed that abandoning CTV1 and reducing CTV2 from the 10mm edge of GTV to 8mm resulted in a 4-year local control rate of 96.6% 28. Table 1 presents 15 studies from multiple countries and regions, covering 5-year overall survival (OS) of NPC under different technical pathways such as 2D-RT, 3D-RT, IMRT, volumetric modulated arc therapy (VMAT), and proton radiotherapy, reflecting the improvement of prognosis with advances in treatment technology 14, 15, 29-41.

## Fine delineation of target area guided by MRI and PET-CT

The superior soft tissue resolution of MRI has unique value in evaluating skull base bone destruction, cavernous sinus, and temporal lobe invasion. MRI/CT fusion has become the standard technique for GTVnx delineation 42. PET-CT can be used to identify metabolically active lymph nodes without significant enlargement on imaging, which helps to confirm the GTVnd boundary and reduce the risk of missed or overexploited target areas 43, 44. At present, it is recommended to combine MRI (structure) + CT (dose planning) + PET (metabolism) three modes to improve the accuracy of target areas.

Ng *et al*. 45 analyzed the value of MRI/CT fusion in tumor delineation and found that the fusion group had an average reduction of 18% in GTV volume in the posterior wall of the nasopharynx, skull base, and other areas compared to the CT group alone, and the recurrence rate also decreased. In terms of PET/CT, Leung TW *et al*. 46 found that 38% of patients with PET positive but CT negative lymph nodes developed regional recurrence during follow-up, suggesting that PET can identify potential lesions in advance and improve the sensitivity of GTVnd delineation. PET/MRI has advantages in anatomical resolution and metabolic activity identification of target area delineation, especially suitable for evaluation after induction chemotherapy 47.

## Standardization and optimization of target area delineation in the cervical lymphatic region

The Robbins system proposed the subdivision of cervical lymphatic regions into zones I-VII in 2003. The 2013 DAHANCA guidelines provided CT axial anatomical boundaries, becoming the most widely used reference currently 48. Lymph node metastasis in NPC is most common in region II (> 80%), followed by regions III and IV, and rare in regions I and VI 49. According to this mode, selective irradiation of the radiotherapy target area is feasible, avoiding full neck irradiation.

Multiple studies 50-54 have supported the safety and efficacy of selective cervical lymphatic irradiation in low-risk populations of NPC from different perspectives (Table 2). Tang LL *et al*. 50 published a nationwide multicenter randomized controlled study, which showed no statistically significant difference in 5-year regional control rate between irradiation of only the upper cervical lymph zone (II-IV zone) and traditional whole neck irradiation (95.7% vs 96.3%, *P* = 0.73), while adverse reactions such as difficulty swallowing and dry mouth significantly decreased. In addition, Hu *et al*. 52 omitted contralateral lower neck irradiation for 52 N1 patients. The results showed that only one patient experienced neck recurrence in the irradiated area, while no patient experienced lymph node recurrence outside the field. Cao *et al*. 55 evaluated the local mass and lymph node metastasis of NPC patients based on PET/MR, and found that only 1.9% of patients experienced skipping lymph node metastasis. The jumping pattern of lymph node metastasis is not common, and participatory radiotherapy may be feasible for cervical lymph node and VIIb levels below the tail edge of the cricoid cartilage. Therefore, selective irradiation is not only safe, but also beneficial for reducing treatment burden and improving QoL, especially for early or low load patients. These studies have suggested that in the population of stages N0-N1 and T1-T3, only irradiation of zones II-IV or II-III can achieve regional control rates similar to full neck irradiation, and selective irradiation is safe and feasible. The toxicity has significantly decreased, with a reduction in the incidence of severe dry mouth, swallowing difficulties, and hearing loss, improving the QoL. Of course, image-based MRI/PET-CT guided assessment of risk areas is the foundation for ensuring the safety of selective irradiation. In the era of whole neck CT, whole neck MRI, PET-CT, it is not necessary to perform long-distance lymphatic irradiation.

## Target delineation strategy after induction chemotherapy

Chemotherapy regimens such as gemcitabine plus cisplatin (GP) and paclitaxel plus cisplatin (TP) can reduce GTV and clear micro metastases 56. Yang *et al*. 11 compared in a prospective clinical trial whether reducing the target volume of IMRT after induction chemotherapy (IC) would affect local control and survival rate. The results showed that reducing the IMRT target volume after IC did not decrease the local control rate and survival rate of locally advanced NPC, but the dose received by normal tissues decreased and the QoL score improved. A prospective phase II study showed that reducing the target volume dose after NPC induced chemotherapy can achieve good local control, limited marginal and field recurrence, and mild late toxicity. Compared with before IC, the target volume after IC significantly decreased, especially for GTVnx and GTVnd 57. A prospective randomized multicenter study showed that reducing the IMRT target area after IC does not affect the local control and survival rate of NPC patients, and can improve their QoL 11. A total of 57 NPC patients underwent IC+CCRT to delineate residual tumor volume as GTV and a radiation dose of 60 Gy. The results showed that the five-year overall survival rate, local recurrence free, distant metastasis free, and progression free survival rates were 82.2%, 85.8%, 87.7%, and 80.3%, respectively 58. A randomized, noninferiority, multicenter phase III clinical study comparing the efficacy of reduced volume (tumor volume after chemotherapy) and traditional volume (tumor volume before chemotherapy) radiotherapy. The results showed that the 3-year local recurrence free survival rate was similar between the two groups (91.5% vs 91.2%). There was no significant difference in FFS, OS, and DMFS. The reduced-volume radiotherapy group showed a significant decrease in both acute and late radiotherapy-related toxicity, along with a notable improvement in QoL 59. The study made the following adjustments to target area delineation: 1) For posterior nasal invasion regression, GTV was delineated based on pre-induction chemotherapy MRI to include the left posterior nasal cavity, and post-chemotherapy MRI excluded the left posterior nostril. 2) For cervical lymph node regression, GTV included the entire level II region on pre-induction chemotherapy MRI, and only residual lymph nodes were included on post-chemotherapy MRI. 3) GTV delineation for skull base bone invasion was based on pre-chemotherapy MRI. 4) Tumor regression areas induced by chemotherapy were included in CTV1. Furthermore, according to the expert consensus published in 2025, for GTVnd with complete remission after IC, dose reduction or narrowing of CTV range can be considered, and it is recommended to combine PET-CT and MRI for accurate evaluation 60. Is the reduction of the primary lesion still based on pre-IC imaging? Does GTVnd disappearance require full dose irradiation? The international consensus of 2025 60 suggests that GTVnx and GTVnd should still be based on IC; After confirming complete remission through PET-CT combined with IC, the CTV1 range can be appropriately reduced. It can be seen that both domestic and foreign experts believe that the delineation of NPC target areas after IC can narrow down the scope, but there is still some controversy over the specific scope of narrowing, which requires us to conduct more large-scale research.

## Adaptive Radiation Therapy (ART)

**Figure 1:**
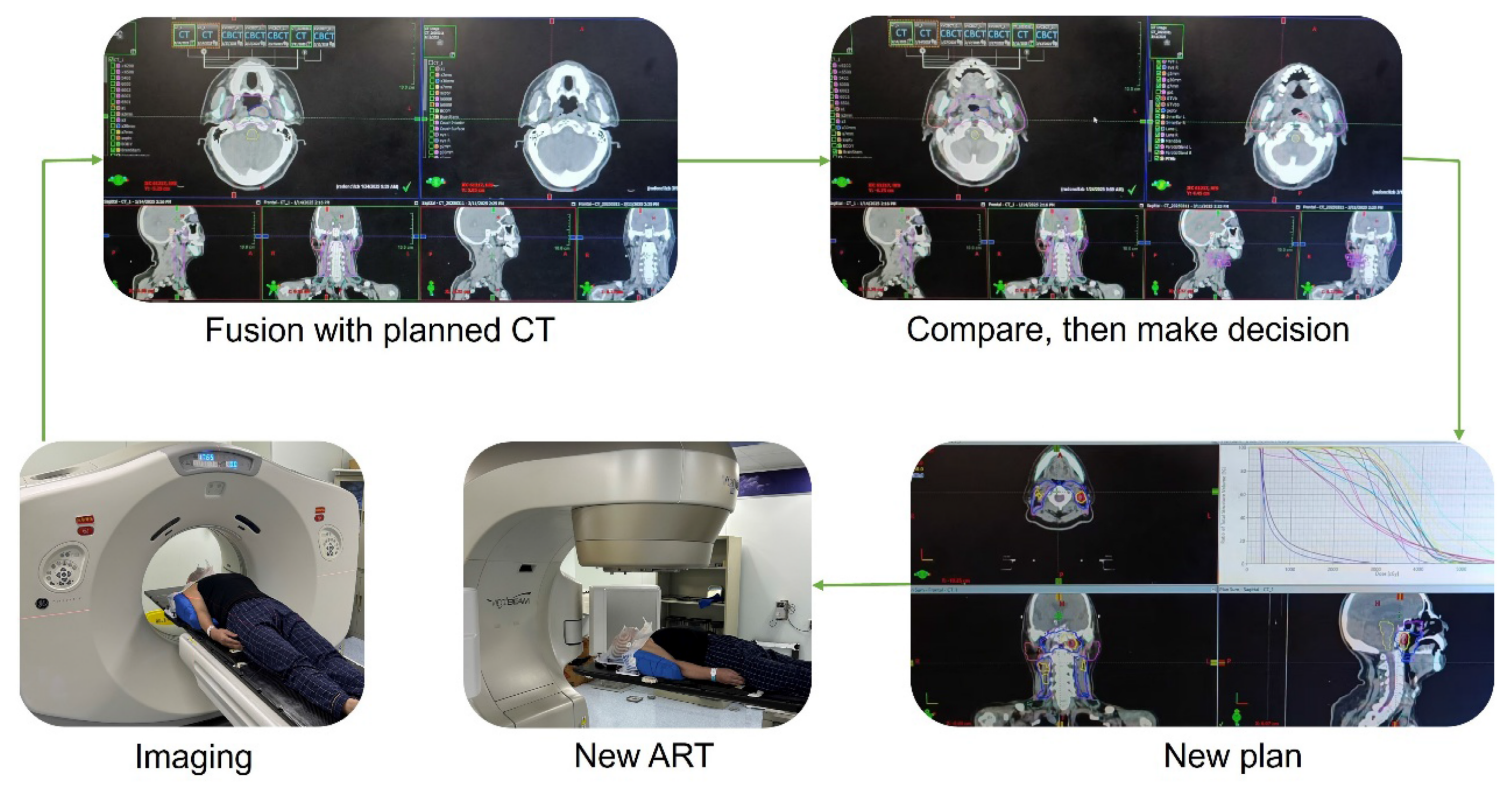
The process of ART. As indicated by the arrow, the first step is to obtain the image; Match the acquired images with the planned CT images; Evaluate the position and dose errors of the target area and OAR, and determine whether ART is needed; If ART is required, readjust the target area and OAR on a new CT to generate a new radiotherapy plan; Finally, use the new plan for treatment.

ART allows for reassessment of target area volume and morphology during treatment (such as tumor shrinkage, weight loss), re planning based on repositioning CT or MRI, reducing toxic side effects, and maintaining target area coverage 61. Daily Cone Beam Computed Tomography (CBCT) positioning can identify changes in body position and volume reduction, which helps ensure treatment accuracy 62. The process of ART is shown in Figure 1. Firstly, CT, MRI, or CBCT images are used, and then the obtained images are fused and registered with the previous planned CT to evaluate the position and dose errors of the target area and OARs, and then, determine whether ART is needed. If the positional deviation is significant, target area correction and plan adjustment will be carried out, and finally a new plan will be used for treatment.

The clinical research on ART in the treatment of head and neck malignant tumors continues to accumulate, showing that it can improve dose consistency and reduce toxic reactions. Zhao *et al*. 63 analyzed 33 NPC patients and found that ART reprogramming improved the 3-year local progression free survival rate of patients with AJCC stage T (3,4) N (x) and alleviated late-stage adverse reactions in patients with large lymph nodes [AJCC stage T (x) N (2,3)]. The head and neck tumor ART study conducted by Bhide SA *et al*. 64 in the UK found that weekly CT monitoring and re planning can reduce the dose to the posterior pharyngeal wall and parotid gland by up to 23%, and improve PTV consistency by 8%. ART is an effective method to improve the precision of radiotherapy implementation. One offline replanning can optimize target coverage, reduce OARs dose, improve tumor control rate, and enhance long-term QoL. ART has shown great potential in NPC, and in the future, combining automatic delineation with artificial intelligence may further improve efficiency and individualization levels.

## Future direction

Using artificial intelligence (AI) and radiomics to assist in target area delineation is expected to achieve better grained individual planning. Combining PET/CT, MRI spectra, metabolic maps, and molecular markers can further enhance the biological accuracy of target area definition. AI can automatically outline and adapt formulaic target areas, reducing manual effort and standardizing the process. Several studies have developed automatic delineation models based on deep learning 65-67, significantly improving the efficiency and consistency of NPC delineation. Imaging omics can predict the risk of local recurrence in advance and provide scientific basis for individualized ART plans 68. Multiple studies have reviewed the systematic application of AI in online re planning, target area prediction, and normal tissue protection, providing a technical blueprint for the development direction of AI driven ART 9, 69, 70.

## Conclusions

The delineation of NPC target areas has evolved from two-dimensional empirical field setting to precise individualized planning based on multimodal imaging. The target area regulation after induction chemotherapy, the rise of ART, and the introduction of AI technology will further promote the intelligence and precision of NPC radiotherapy. The future target area definition should integrate multidimensional information such as imaging, molecular markers, and immune responses to achieve a new pattern of radiotherapy that is "precise definition, individual regulation, and real-time adaptation".

## Author Contributions

Xiaojing Yang: Writing - review & editing; Lijun Liu: Imaging; Huicai Zhang: Imaging; Hanru Ren: Tabulating and auditing; Jie Fu: Writing - review & editing.

## Figures and Tables

**Table 1:** Comparison of 5-year OS of NPC patients under different periods and techniques.

Radiotherapy techniques	Author, Year	Case	5-year OS	Note
2D-RT	Waldron *et al*., 2003 14	94	88%	The Princess Margaret Hospital, 1993-1997
2D-RT	Lee *et al*., 2005 15	1282	62%	Hong Kong, 1980-1995
2D-RT+CCRT	Wei *et al*., 1998 29	298	63.4%	Cisplatin synchronous chemotherapy
3D-CRT	Tang *et al*., 2006 30	87	88.2% (3-year OS)	State Key Laboratory of Oncology in South China, 2001-2004
3D-CRT	Yi *et al*., 2006 31	905	76.1%	Beijing, 1990-1999
IMRT	Kam *et al*., 2007 32	193	83%	The Chinese University of Hong Kong, forward-looking
IMRT	Su *et al*., 2012 33	198	97.3%	2001-2008, patients in stages T1-T2
IMRT	Lee *et al*., 2009 34	74	85%	MSKCC, USA
IMRT ± CCRT	Yi *et al*., 2014 35	333	79.4%	locoregionally advanced NPC, 2001-2009
IMRT + CCRT	Chen *et al*., 2013 36	508	84.0%	Sun Yat-sen University Cancer Centre, patients in non-metastatic stage III or IV (except T3-4N0)
IMRT + IC + CCRT	Sun *et al*., 2016 37	480	90%	RCT, patients in stage III-IVB (except T3-4N0)
IMRT + IC + CCRT	Zhang *et al*., 2020 38	541	89.5%	Tiggio/paclitaxel induction regimen
VMAT + IC	Liu *et al*., 2018 39	139	57.5%	patients in stage II-IVB
IMPT	Lewis *et al*., 2016 40	10	88.9% (2-year OS)	MD Anderson Cancer Center
IMPT	Li *et al*., 2021 41	77	100% (3-year OS)	Memorial Sloan Kettering Cancer Center

OS: overall survival; NPC: nasopharyngeal carcinoma; 2D-RT: two-dimensional radiotherapy; 3D-CRT: three-dimensional conformal radiotherapy; IMPT: intensity modulated proton therapy

**Table 2:** Selective Neck Lymphatic Region Radiation vs. Whole Neck Radiation.

Author (year)	Stage	Groups	5-year regional control rate	Grade III dry mouth rate	Conclusion
Tang LL *et al*., 2022 50	N0-N1	II-IV vs whole neck	95.7% vs 96.3%	9.4% vs 18.2%	RCT, Selective irradiation does not reduce control rate
Seol KH *et al*., 2016 51	T1-4N0-3M0	level Ib, IV, and Vb nodes omitted	87.1% (3-year)	-	No nodal failure
Hu W *et al*., 2013 52	N1	contralateral lower neck omitted	94.3% (3-year)	-	Omitted the contralateral lower neck in a N1 stage NPC was safe and practicable
Li JG *et al*., 2013 53	T1-4N0M0	upper neck irradiation vs. whole neck irradiation	89.8% vs 89.3%(3-year)	1.3%vs 2.7%	Prophylactic upper neck irradiation is sufficient for patients with node-negative NPC
Chen JZ *et al*., 2013 54	T1-4N0-1M0	elective neck irradiation to level IV and Vb nodes	95.6%	17.9% (> Grade II)	Elective neck irradiation to level IV and Vb nodes in N0-1 NPC is safe
